# Virtual Sensory Feedback for Gait Improvement in Neurological Patients

**DOI:** 10.3389/fneur.2013.00138

**Published:** 2013-10-14

**Authors:** Yoram Baram

**Affiliations:** ^1^Computer Science Department, Technion – Israel Institute of Technology, Haifa, Israel

**Keywords:** sensory feedback, gait improvement, virtual reality, closed-loop gait regulation, sensory-motor control

## Abstract

We review a treatment modality for movement disorders by sensory feedback. The natural closed-loop sensory-motor feedback system is imitated by a wearable virtual reality apparatus, employing body-mounted inertial sensors and responding dynamically to the patient’s own motion. Clinical trials have shown a significant gait improvement in patients with Parkinson’s disease using the apparatus. In contrast to open-loop devices, which impose constant-velocity visual cues in a “treadmill” fashion, or rhythmic auditory cues in a “metronome” fashion, requiring constant vigilance and attention strategies, and, in some cases, instigating freezing in Parkinson’s patients, the closed-loop device improved gait parameters and eliminated freezing in most patients, without side effects. Patients with multiple sclerosis, previous stroke, senile gait, and cerebral palsy using the device also improved their balance and gait substantially. Training with the device has produced a residual improvement, suggesting virtual sensory feedback for the treatment of neurological movement disorders.

## Introduction

Neurological disorders, such as Parkinson’s disease (PD), multiple sclerosis (MS), previous stroke (PS), senile gait (SG), and cerebral palsy (CP), often entail mobility impairment. Traditionally, gait rehabilitation, whether by means of physiotherapy or pharmacological treatment, has focused on improvement of muscle strength and reduction of spasticity (1–3). However, the main causes of motor impairment in such cases appear to lie in dysfunctional brain structures and neural information pathways. In particular, it has been suggested that PD patients suffer from deficient internal cue production (4–6). Visual feedback cues in the form of transverse lines marking on the ground have been found to improve the walking abilities of patients with PD (7, 8). Moreover, deficits in the functional neuroanatomy underlying gait in PD patients were found to be compensated by visual cues (9). Specifically, the right lateral pre-motor cortex, which is mainly regulated by cerebellar inputs, was activated to a greater extent in PD patients than in age-matched healthy individuals by visual transverse lines. On the other hand, healthy individuals activated mainly the supplementary motor area (SMA), which was under-activated in PD patients. It appears, then, that visually enhanced gait employs different brain pathways in PD patients compared to healthy individuals. It has been suggested that external sensory cues help patients with PD switch from one movement component of a sequence to the next, bypassing the defective internal trigger of the SMA (10, 11). It seems plausible that the concept of sensory bypass of deficient brain structures can apply to other categories of neurological disorders.

Early attempts to improve gait by artificially generated auditory and visual signals have produced open-loop systems which impose sensory signals, generated by an external source, not affected by the patient’s own motion, such as fixed-velocity (treadmill-like) visual cues or rhythmic (metronome-like) auditory cues. While such strategies have been found to produce gait improvement in several studies (12–18), others have reported a need for constant vigilance and attention strategies to prevent reversion to impaired gait patterns caused by repetitive stimuli (11), confirming the role of predictive novelty and saliency in dopamine reward (19). Moreover, open-loop systems are known to be inherently inaccurate and unstable (20), which, in the present context, would be manifested by the patient “falling out of sync” with the repetitive sensory stimulus. A comparison of open-loop visual cuing by technological means to transverse lines marking on the ground (21) has found the first to have a marginal effect and the second to have a significant positive effect on walking parameters in PD patients. The advantage of closed-loop over open-loop control of arm motion has been noted (10). More recently, closed-loop sensory feedback strategies have been implemented in such specific motor control functions, related to locomotion, as stationary balance (22), planar pelvis and trunk movement (23), step symmetry (24), knee hyperextension (25), and partial weight-bearing (26). Yet, closed-loop virtual sensory feedback of whole-body forward movement in locomotion, as presently reviewed, does not appear to have been implemented, tested, or analyzed in other works.

## Closed-Loop Sensory Feedback Gait Control

**Figure 1:**
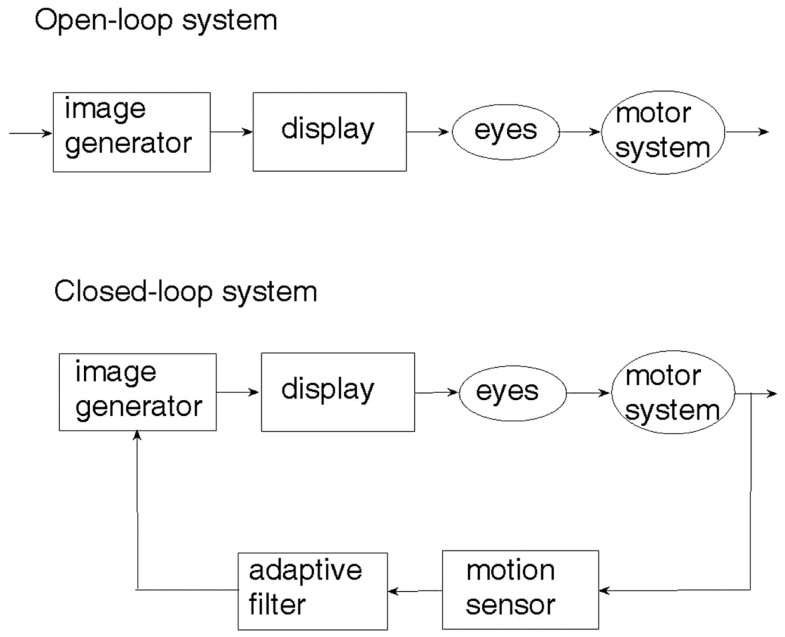
**Closed-loop vs. open-loop sensory-motor control system**.

An examination of the natural sensory-motor control system underlying human locomotion with respect to a visual scenery (27) reveals that it is the physical motion of the body which generates the visual cue and not the other way around. This seemingly obvious observation is crucial to understanding the difference between open-loop and closed-loop sensory control of locomotion. The two control paradigms are illustrated in Figure 1. In the open-loop system, a visual cue is generated artificially and fed through the eyes to the brain, which may or may not activate the limbs so as to respond to the visual cue. On the other hand, an artificial realization of the natural sensory-motor control system is the closed-loop feedback system, where the generation of the visual cue is controlled and regulated by the body movement caused by locomotion. The motion of the visual cue is matched to that of the body. When there is no motion of the body, there is no visual cue.

**Figure 2:**
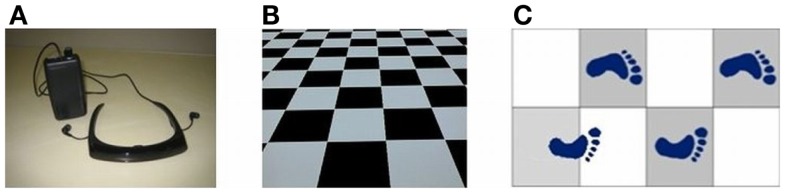
**(A)** Sensory feedback apparatus. **(B)** Glide-symmetric tile pattern displayed by apparatus. **(C)** Gait regulation by visual feedback.

Depicted in Figure 2A, a sensory device, employing body-mounted inertial sensors, generates earth-stationary visual cues (28). The checkerboard tiles geometry of the visual display, Figure 2B, matched to the glide-reflection symmetry of human locomotion (29), regulates the motor task, producing an even (glide-reflection symmetric) gait pattern, better balance, and safer, more efficient mobility. Even if the resulting gait pattern is not perfectly matched to the visual tile pattern, improvement in that direction translates into improvement in gait. Figure 2C illustrates the transition from uneven to even gait resulting from such visual feedback regulation (a short first step is followed by longer, more even steps). In addition to the visual feedback cue delivered by the display, the device also produces an auditory feedback cue in the form of a clicking sound delivered through earphones in response to every step taken by the patient. In contrast to open-loop, metronome-like devices, which attempt to impose a walking pace on the patient by a constant auditory cue, the feedback device produces an auditory cue matched to the walking pattern. A balanced steady walk will generate a rhythmic auditory cue. Any deviation from such a gait pattern will result in a deviation from the auditory rhythm and will be corrected by a change of gait in a feedback fashion. The head-mounted display and earphones bring the sensory feedback signals closer to the sensors – the eyes and the ears, making the sensory effect more pronounced, easier to follow and to learn. An open-loop capability, producing constant movement of the visual cue and a constant rhythmic auditory cue, was also added to an early version of the device for experimental comparison purposes.

## Medical Studies

Medical studies, described below and summarized in Table 1, were performed during different stages in the development of the concept, the apparatus, and the medical assessment methods. Consequently, these studies varied not only in the different research groups and their focus on different neurological disorders, but also in the test parameters. Some of the studies tested visual feedback only, some auditory feedback only, and some both visual and auditory feedback. Some of the tests examined on-line (device on) performance only, some extended to examination of short-term (a few minutes) residual effects, and some examined long-term (a few weeks) residual effects as well. The results of the different studies also differed in their methods of statistical representation. Yet, most of the studies shared some common features, particularly the following three steps:

Step 1: baseline performance. The patient was verbally instructed to walk “normally” without the device along a straight track of 10 m. The time to complete the track and the number of steps were recorded for calculation of baseline walking speed (BWS) and stride length.
Step 2: on-line training and performance. The device was placed on the patient and turned on. The patient was instructed to walk along the 10-m track for the purpose of training. Patients training with visual feedback were asked to imagine, while walking, stepping on the tiles. They were told that stepping on tile boundaries was allowed, and that there was no particular order of black or white tiles that needed to be kept with respect to the stepping sequence. Patients training with auditory feedback were asked to maintain, by controlling their gait pattern, a rhythmic auditory cue. The track walking was repeated twice for training and four more times for measurement and recording of on-line walking speed and stride length.
Step 3: residual effect. The device was taken off the patient, who was given a 20-min break. After the break, the patient was instructed to walk the 10-m track without the device. Walking speed and stride length were recorded four times and averaged. The purpose of this stage was to measure the residual short-term effect of training with the sensory feedback cue.

**Table 1:** **Clinical test settings**.

Study	Disease	Number of patients	Patient condition	Feedback channel	Feedback modality	Training location	Effect measured	Typical change
In-clinic comparison of open and closed-loop strategies	PD	14	Off	V	O	C	OL	13.8%
In-clinic comparison of open and closed-loop strategies	PD	14	Off	V	C	C	OL	25.6%
At-home training with joint visual and auditory feedback	PD	13	On	VA	C	H	OL	17.9%
At-home training with joint visual and auditory feedback	PD	13	On	VA	C	H	LTR	17.1%
Gait initiation and the significance of prior instructions and training	PD	47	On	V	C	C	GI	6.2%
Long-term effects on PD patients with “on”-predominant freezing of gait	PD	13	On	VA	C	H	UPDRS	35.4%
Visual feedback	SG	20	On	V	C	C	OL	6.3%
Level of education effect	SG	20	On	V	C	C	EL	6.7%
Visual feedback	PS	6	On	V	C	C	OL	13.2%
Visual feedback	MS	16	On	V	C	C	OL	13.4%
Visual feedback	MS	16	On	V	C	C	STR	24.4%
Auditory feedback	MS	16	On	A	C	C	OL	12.8%
Auditory feedback	MS	16	On	A	C	C	STR	18.7%
Visual cue geometry effect	MS	16	On	V	C	C	VCG	21.0%
Visual feedback	CP	10	On	V	C	C	STR	21.7%
Auditory feedback	CP	10	On	A	C	C	STR	25.4%

Patient condition: Off, without medication for 12 h; On, on regular medication schedule. Feedback channel: V, visual channel; A, auditory channel; VA, combined visual and auditory channels. Feedback modality: O, open-loop; C, closed-loop. Training location: C, clinic; H, home. Effect measured: OL, on-line; STR, short-term residual; LTR, long-term residual; GI, gait initiation; UPRDS, unified Parkinson’s disease rating scale; EL, education level; VCG, visual cue geometry. Typical change: percentage improvement in walking speed compared to baseline, except where corresponding to GI, UPDRS.

The results were then averaged across all patients and the average change due to device use was calculated along with the significance parameter *p*.

While there are several on-going studies on the effects of the GaitAid virtual sensory feedback device, performed by different research groups concerning different disorders and different issues of interests, we provide an account of earlier studies, detailed in previous publications.

### Parkinson’s disease

#### In-clinic comparison of open and closed-loop strategies

A clinical study comparing the on-line effects of visual cues in open-loop and closed-loop configurations on PD patients off their regular medication (30) has found that patients who used the open-loop system improved their gait on average by 13.8% (*p* = 0.230) in walking speed and by 15.0% (*p* = 0.056) in stride length. Two of the patients went into freezing midway when using the open-loop system. The high *p* values indicate low likelihood of the improvement results being attributable to a specific cause. Patients who used the closed-loop system improved their gait on average by 25.7% (*p* = 0.001) in walking speed and by 30.8% (*p* = 0.0085) in stride length. None of the patients experienced freezing when using the closed-loop system. This study revealed that the gait parameters which are most sensitive to anti-Parkinson medication (31), namely, walking speed and stride length, can also be manipulated, to a similar extent and without some adverse effects, by a closed-loop display of virtual visual cues. These parameters have also been reported to be improved by pallidotomy [brain surgery (32)], however, a more recent study has shown adverse effects of deep brain stimulation (DBS) of the globus pallidus internus (GPi) in PD patients with dystonia (33).

#### At-home training with joint visual and auditory feedback

Clinical testing before and after 2-week at-home training with joint visual and auditory feedback (34) found that average improvement with device on was 17.9% (*p* = 0.006) in walking speed and 13.1% (*p* = 0.004) in stride length. Residual improvement in walking without the device was 17.1% (*p* = 0.0004) in walking speed and 12.4% (*p* = 0.003) in stride length. Residual improvement of two thirds of the patients was at least 20% (*p* < 0.03) in either walking speed or stride length or both. Improvement in FOGQ (35) was 14.5% (*p* = 0.02). This shows that although the immediate improvement with device use, or immediately following such use, was somewhat higher (30), residual improvement was sustained at least for a few days following training.

#### Gait initiation and the significance of prior instructions and training

A study of visual feedback without prior instructions or prior training (36) has shown a decrease in the average time of first-step initiation (−6.2%), with smaller changes in subsequent average walking speed (−0.8%) and cadence (−1.8%). The improvement in step initiation by device use suggests a role for predictive salience and dopamine reward in movement (19). A comparison of the present study to previous studies, in which patients were given prior instructions and training, resulting in the sustainment of an improved gait pattern, suggests that predictive salience and dopamine reward are enhanced by prior instructions and training.

#### Long-term effects on PD patients with “on”-predominant freezing of gait

A study on the long-term effects of training with sensory feedback has examined PD patients with “on”-predominant freezing of gait (37). Of the 13 initial patients only 2 completed the study, which was attributed to severe burden of the disability and the fragility of these patients, limiting the opportunities to fulfill the required daily training sessions and preventing their return for the scheduled study visits. The single patient who was documented showed a sustained improvement in the UPDRS-III (38) and the FOGQ (35) measures, following 4 weeks of at-home training (UPDRS-III: 24 at baseline, 15.5 at 12 weeks; FOGQ: 16 at baseline, 13 at 12 weeks). Benefits were renewed after a “booster” training once the residual gait improvement weaned at about 16 weeks post-training. Yet, the high dropout rate did not support generalization of these results.

### Senile gait

#### Gait improvement

A study of randomly selected old-age home residents suffering from lower-body Parkinsonism, or SG, but without PD (39) showed that, in patients with baseline performance above the median, the average on-line improvement when using visual feedback was considerably higher (6.31 ± 12.59% in walking speed and 6.41 ± 11.11% in stride length) than in patients with baseline performance below the median (−0.72 ± 22.75% in walking speed and 3.39 ± 11.26% in stride length). This stands in sharp contrast to the results obtained for patients with PD, whose gait improvement was inversely correlated with baseline performance.

#### Level of education effect on SG improvement

The same study (39) found that, for patients with a maximum of 8 years of study, average improvement in walking speed was −8.83 ± 23.81 and −4.67 ± 15.30% in stride length. For patients with 12 years of study, average improvement was −1.85 ± 26.83% in walking speed and 3.82 ± 9.75% in stride length. For patients with 20 years of study, average improvement was 6.75 ± 0.49% in walking speed and 14.55 ± 12.66% in stride length. As might have been expected, the ability to make use of sensory feedback information appeared to be positively correlated with cognitive abilities. Conversely, improved performance by sensory feedback may be regarded as a relevant measure of cognitive function in the present context.

### Previous strokes

Two thirds of the patients with SG also suffering from PS (39), using on-line visual feedback as specified in steps 1 and 2, improved their walking speed or stride length or both by more than 10%. While patients with left-hemisphere vascular accident improved their gait, patients with right-hemisphere vascular accident did not improve. In patients with baseline performance above the median, improvement was considerably higher (13.2 ± 6.0% in walking speed and 16.6 ± 4.7% in stride length) than in patients with baseline performance below the median (−9.9 ± 27% in walking speed and −7.7 ± 12.3% in stride length). The visual feedback cues did not improve gait in patients with vascular risk factors but without history of PS. As the gait improvement in patients with PS was more pronounced than in patients with SG but without PS, so was the contrast between these patients and patients with PD in the correlation between the level of improvement and baseline performance.

### Multiple sclerosis

In contrast to patients with PD, SG, or PS, who are predominantly of advanced age, patients with MS range from teen-agers to mid-agers.

#### Visual feedback

A study of the effects of visual feedback on patients with MS (40) found that patients whose BWS was below the median showed an average on-line improvement of 13.46% in their walking speed when using the visual feedback channel of the GaitAid device, while patients whose BWS was above the median improved their walking speed by 1.47%. The average short-term residual improvement in walking speed was 24.49% in patients with BWS below the median and 9.09% in patients with BWS above the median. Similar results were obtained for improvement in stride length. These trends are consistent with those found in patients with PD. No gait improvement was found in age-matched controls using visual feedback.

#### Auditory feedback

A study of the effects of auditory feedback on patients with MS (41) showed an average improvement of 12.84 ± 18.74% on-line and 18.75 ± 18.53% residually in walking speed. Average improvement in stride length was 8.30 ± 11.87% on-line and 9.93 ± 9.46% residually. No gait improvement was found in age-matched controls using auditory feedback.

#### Visual cue geometry

A study aimed at comparing the effects of gait training with distinct glide-reflection symmetry (checkerboard tiles) visual feedback cues, to the effects of training with visual cues with no distinct symmetry [earth-stationary transverse lines, as used in early studies (7, 8)] was performed on subjects with gait disorders due to MS (42). It found that the average improvement in the group using the transverse lines was 7.79 ± 4.24% in walking speed and 7.20 ± 3.92% in stride length. The average improvement in the group using the visual cue of checkerboard tiles was 21.09 ± 18.39% in walking speed and 12.99 ± 1.72% in stride length. This shows that matching the visual cue pattern to the glide-reflection symmetric pattern of human locomotion results in significant additional improvement.

### Cerebral palsy

Cerebral palsy has predominantly pre-natal causes and is symptomatically addressed at a young age. A study of patients with gait disorders due to CP (43) found that, for patients training with visual feedback, the short-term residual improvement was 21.70 ± 36.06% in the walking speed and 8.72 ± 9.47% in the stride length. For patients training with auditory feedback, the short-term residual improvement was 25.43 ± 28.65% in the walking speed and 13.58 ± 13.10% in the stride length. Age-matched controls who trained with either visual or auditory feedback showed no improvement in gait. The relatively large standard deviations in the results may be attributed to the very diverse nature of the disorder and the subsequent disabilities.

### Medical studies summary

The reviewed medical studies are summarized in Table 1.

It can be seen that, as indicated by the last column of Table 1, all these medical studies show positive effects of sensory feedback on gait in patients with the neurological disorders under consideration. Yet, any comparison between these results must take into account the differences between the disorders, the testing conditions and the measures used, as indicated in the specific subsections, and, in further detail, in the cited references. For instance, the percentage improvements in gait initiation time (GI) and in UPDRS bring into light different aspects of gait improvement in PD patients, but cannot be used to compare the benefits for MS patients to those for PD patients. It should also be noted that while these studies employed various versions of monocular and binocular displays, on the one hand, and either or both visual and auditory feedback channels, the results do not necessarily generalize to all forms of sensory feedback, which may present additional benefits or drawbacks.

## Discussion

We have reviewed the use of sensory feedback for improving gait in movement disorders patients. While certain studies have found open-loop sensory stimulation to result in balance and gait improvement, others have raised questions as to the effectiveness of monotone sensory cues, which, lacking the predictive novelty and saliency associated with dopamine reward, require constant vigilance and attention strategies, and instigate freezing in Parkinson’s patients who fall out of sync with the sensory stimuli. Realizing that the natural sensory-motor stabilization effect is produced by the sensory cues generated by motion in a stationary environment, we created a closed-loop augmented reality device which produces earth-stationary visual and auditory cues in response to the patient’s own motion. The device has been found to improve gait in patients with a variety of neurological disorders, maintaining patient’s safety and well-being, without the adverse effects associated with medication and deep brain surgery. Neurological patients are often subject to high levels of fragility and fatigue, which may affect their ability to sustain long periods of physical training in general, and training with sensory feedback in particular. We did not encounter, however, significant levels of such effects in our studies, where particularly disabled patients were excluded. With the exclusion of patients with very low or very high level of impairment, the level of improvement is normally related to the level of impairment. Training with the device has been found to have short-term and long-term residual improvement effects, suggesting virtual sensory feedback as a treatment modality for neurological movement disorders. While, due to conceptual and technological developments, the device took different forms, the concepts tested were quite general (e.g., visual feedback, auditory feedback, and combined visual and auditory feedback). Moreover, for the same concept (e.g., on-line visual feedback in PD patients), the results were quite consistent. It is therefore believed that if other devices, based on the same concepts, become available, they will produce similar results.

As the clinical studies performed on a variety of patient populations with different neurological impairments were of a preliminary nature, future studies, involving larger patient cohorts under streamlined controlled conditions, should investigate the same and other aspects of such treatment in greater detail. The possibility of fitting the sensory feedback device to individual patient limitations and needs should be explored. Long-term treatment programs should be developed and tested in clinic, home and, possibly, a variety of natural environments. The integration of sensory feedback components of specific motor tasks associated with gait and balance, such as head, pelvis, trunk, and knee movement, in a comprehensive motor rehabilitation program, should also be investigated. Mobile technology advancement, improving vision, hearing and sensing, should be adopted to improve device utility and effectiveness.

## Conflict of Interest Statement

The author is the developer of the sensory feedback device used in the reported medical studies.
